# Neural Bases for Individual Differences in the Subjective Experience of Short Durations (Less than 2 Seconds)

**DOI:** 10.1371/journal.pone.0054669

**Published:** 2013-01-16

**Authors:** Jason Tipples, Victoria Brattan, Pat Johnston

**Affiliations:** 1 Department of Psychology, University of Hull, Hull, United Kingdom; 2 Department of Psychology, University of York, York, United Kingdom; University of Milan, Italy

## Abstract

The current research was designed to establish whether individual differences in timing performance predict neural activation in the areas that subserve the perception of short durations ranging between 400 and 1600 milliseconds. Seventeen participants completed both a temporal bisection task and a control task, in a mixed fMRI design. In keeping with previous research, there was increased activation in a network of regions typically active during time perception including the right supplementary motor area (SMA) and right pre-SMA and basal ganglia (including the putamen and right pallidum). Furthermore, correlations between neural activity in the right inferior frontal gyrus and SMA and timing performance corroborate the results of a recent meta-analysis and are further evidence that the SMA forms part of a neural clock that is responsible for the accumulation of temporal information. Specifically, subjective lengthening of the perceived duration were associated with increased activation in both the right SMA (and right pre-SMA) and right inferior frontal gyrus.

## Introduction

Individuals differ in the ability to estimate time intervals [1] and perceive beat information in music [2]. Nonetheless, despite growth in research into the neural mechanisms responsible for timing [3], individual differences in the neural substrates of the timing of intervals less than 2 seconds in duration remains a relatively unexplored topic of research. Here, we present data that shows that individual differences in interval estimation predict neural activation in regions thought responsible for the accumulation of temporal information.

Attempts to isolate the neural substrates for time perception are made difficult by the fact that time perception most likely engages numerous cognitive processes. Consequently, neural activation studies may either falsely attribute activation to timing when it reflects the operation of a different process or fail to record an effect of timing because activation is masked by a separate process (or processes). One solution is to isolate variability in neural activation through experimental control or manipulation for example, by testing for the effects of working memory demands [4]–[6] and motor processes [7].

A separate but complementary approach is to test ideas derived from information processing models of interval timing [8], [9] that specify the operation of a dedicated internal clock. Internal clock models of interval timing typically include 1) an arousal-sensitive pacemaker that emits units of time (or pulses) 2) a switch that controls the flow of pulses and 3) an accumulator where perceived time is calculated based on the total number of counted units. The counted time is subsequently compared to a reference duration that is held in working memory. A simple prediction that derives from this model is that neural activity will grow linearly in the region that is responsible for the accumulation of time. Drawing on this model, two studies [10], [11] have tested for the operation of an accumulator, by attempting to isolate increases in neural activity linked to linear increases in the duration of a timed interval. For example, an innovative aspect of one study [10] was the selection (through pilot testing) of pairs of sample (S1) and probe (S2) stimuli that varied parametrically in duration (from 300 to 1500 ms) but were nonetheless, orthogonal with respect to the difficulty associated with comparing the S1/S2 pairs. Using this design, the authors were able to isolate increased brain activity due to increases in time from activity associated with the difficulty of comparing durations. The results showed that linear increases in (S1) duration were associated with increased activation in anatomically defined regions of interest including the left inferior frontal gyrus, SMA and superior temporal cortex.

A separate study [11] examined neural activity during the encoding and reproduction of multi-second durations that ranged between 5 and 16.82 seconds. During the reproduction phase of the task, there was a positive correlation between interval duration and neural activation in the SMA, superior frontal sulcus, precentral gyrus (primary motor cortex) and in agreement with the proposed role of basal ganglia in timing [12], the right caudate nucleus. During the encoding phase, activity in the left dorsolateral prefrontal cortex was negatively correlated with interval duration. Overall, the results of both studies support the idea that the SMA plays a key role in, but is not uniquely responsible for, temporal accumulation.

Testing for an association between individual differences in timing and neural activity offers another way of testing specific hypotheses derived from internal clock models of timing. So far, the majority of fMRI studies of time perception have averaged across the data of each individual to test for group differences in timing and subsequently have not tested for an association between individual differences in timing and neural activation due to timing. There are a number of reasons why testing for such an association makes sense. First, if both behaviour and neural activation reflect the same process then an association between individual differences in timing and neural activation supports the idea that neural activation during timing tasks is a measure of time perception rather than reflecting unrelated processes. In other words, a correlation offers corroboratory evidence for the role of specific neural regions involved in timing. Second, including individual differences may be necessary to reveal timing processes. For example, a correlation between individual differences and neural activation may be present when group main effects are absent [12]. Third, time perception is linked to genetic differences between individuals. Specifically, genetic polymorphisms of dopamine [13] and serotonin [14], [15] receptors have been linked to timing. Fourth, individual differences may predict differences in neural activation at different stages of timing (e.g., at an encoding stage but not a decision making stage) and therefore, increase the sensitivity of fMRI to isolate effects of timing processes through increased statistical power. Finally, time estimation is sensitive to arousal [16] that is most likely increased for some but not all individuals during fMRI scanning. Moreover, given that individual differences in timing exist (see for example; [1]) it makes sense to include individual differences in timing performance as a predictor of neural activation.

A number of researchers [17]–[19] have argued for distinct neural mechanisms for the timing of relatively brief durations (e.g., typically less than 1 second) compared to long durations (e.g., typically greater than 1 second) and therefore, it makes sense to consider the range of durations used to study the effects of individual differences in neural activation due to timing. For studies that have used relatively long durations (greater than 2 seconds), recent findings support the idea that individual differences contribute to neural activation due to timing. Specifically, one research group [20], [21] reported positive correlations between both behavioural estimates of time and personality measures (of impulsivity) and neural activation during the timing of 9 and 18 second intervals. In separate research, Gilaie-Dotan and colleagues [22] recorded a positive association between gray matter volume in both the right posterior lateral sulcus (encompassing the primary auditory and secondary somatosensory cortices) and parahippocampal gyrus and an individual's ability to discriminate longer durations of 12 s (but not shorter ones of 2 s) regardless of whether they were presented in auditory or visual modalities. These different strands of research support the potential of including individual differences as predictors or covariates in studies of time perception.

Results from studies of timing for relatively brief durations (less than 2 seconds) also show that individual differences in timing predict timing related neural activity. For example, in the study discussed previously [10], individual differences in the accuracy of comparing probe and sample durations was associated with linear increases in activity in two pre-defined Regions of Interest (ROIs), namely, the left inferior frontal regions including the left pars triangularis and the right frontal operculum. To re-iterate, the experimental design of this research ensured that comparison difficulty was orthogonal to parametric variations in duration and consequently, this aspect of the experiment enabled the authors to attribute this activation to comparison difficulty rather than the accumulation of time. Other event-related fMRI studies have recorded an association between individual differences in timing and neural activation that they have attributed to the accumulation of time rather than the process of comparing durations (comparator activity). For example, in one event-related fMRI study [23], increased accuracy during the encoding of temporal intervals but not during the comparison of intervals predicted activity in the putamen. The correlation was not found on a control (colour discrimination) task that used identical stimuli. A separate study [24], that was specifically designed to examine individual differences in neural activation due to timing also recorded an association between individual differences in timing and increases in activity in the putamen. Specifically, individual differences in temporal reproduction times for the encoding of visual, but not auditory signals, predicted increased activity in the right putamen, the right mid-insula and the right mid superior temporal cortex. Overall, the results of these studies of timing for durations less than 2 seconds offer further support for the idea that individual differences in timing predict brain activation linked to timing and consequently, are useful for isolating components of the putative internal clock.

Although there has been clear progress in describing the neural correlates of individual differences in timing, very few studies have attempted to study the association between neural activation and standard psychophysical indices of timing such as the Bisection Point (BP; duration at which participants respond equally often ‘short’ and ‘long’). It may be profitable to design fMRI studies so that the BP and other indices such as the Weber ratio can be calculated because 1) they have been calculated extensively in behavioural research and 2) they can be calculated for each individual and therefore, used to assess the role of individual differences in time perception. One study [25] that did calculate the BP for each individual recorded a positive correlation between the BP and activation in the right parahippocampus. Here, we extend this approach by testing for a correlation between timing performance as indexed by BP and activity within the network of structures identified in a recent review [3] and meta-analysis [26] as typically showing increased activation (averaged across individuals) for timing vs. control performance. This network includes the SMA, the basal ganglia (including the putamen and the caudate nucleus), the ventral premotor cortex (in the region around the frontal operculum), and the cerebellum. The right inferior gryrus and SMA are of particular interest because a recent meta-analysis showed that these areas are consistently activated across a range of timing tasks used in fMRI research. Indeed, there is both fMRI [23], [27], [28] and electrophysiological research [29] evidence that supports the idea that SMA may act as an accumulator mechanism. To re-iterate, if the SMA acts as an accumulator, then for tasks that require perceptual timing (e.g., discrimination of long and short intervals) greater increases in activity are expected for those individuals who perceive more time as having passed. For tasks that require individuals to reproduce an interval, relatively greater accumulation is predicted to lead to shorter estimates of time because, if the clock runs faster in these individuals, then more time will be perceived as having passed and consequently reproduction of the interval will be terminated sooner (relative to those individuals for whom the internal clock runs more slowly).

In the current research we tested for an association between individual differences in timing performance and neural activation due to time estimation using a modified version of the Temporal Bisection Task (TBT) that was originally used to examine perceptual timing in non-human animals [30] and later adapted for use with humans [31], [32]. In the version of the TBT used here [33], participants were asked to judge whether face stimuli were displayed for a duration that was more similar to either a short or long duration that they previously learnt to recognise in a separate learning phase (that took place outside the scanner). Face stimuli were used because they 1) readily attract attention [34] and therefore, allow observers to efficiently start interval timing [35] and 2) have multiple attributes (e.g., sex, age, identity, emotion) that can be judged in a control task. Furthermore, recent research has shown that a version of the task used here that used face stimuli is sensitive to individual differences [36], [37]. To maximise power to detect differences we chose a mixed or part blocked design in which participants were required to alternate between judging facial gender and the duration of the face stimuli (duration varied within blocks; see design).

In sum, our objective was to test for modulation of timing-specific neural activation by individual differences in time estimation. The first hypothesis is that there will be greater activation in neural regions previously shown to be responsible for time perception, including the SMA, basal ganglia and right inferior frontal gyrus when participants judge the duration of faces compared to when they judge the sex of the faces. The second hypothesis is derived from previous research [23], [28], [29] that has reported evidence supporting the idea that the SMA is responsible for the accumulation of units of time. Specifically, the prediction is that the subjective experience of time (indexed by the BP) will predict linear increases in activation in the SMA.

## Materials and Methods

### Participants

All participants were right handed and had normal to corrected-to normal vision. Written consent was obtained for all participants, and the study was approved by the York Neuroimaging Centre (YNiC) Ethics Committee.

### Imaging parameters

All experiments were performed using a GE 3 Tesla HD Excite MRI scanner at the York Neuroimaging Center at the University of York. A Magnex head-dedicated gradient insert coil was used in conjunction with a birdcage, radio-frequency coil tuned to 127.4 MHz. A T2*-weighted gradient-echo EPI sequence was used to collect a series of 305 brain volumes (TR  = 3 s, TE  = 25 ms, FOV  = 28×28 cm, matrix size  = 128×128, 39 axial slices, slice thickness  = 3 mm). After the functional scan, a high-resolution T1-weighted structural volume was acquired using 3D FSPGR (Sagittal Isotropic 3D Fast Spoiled Gradient-Recalled Echo) pulse sequence (TR  = 8.03 sec, TE  = 3.07 sec; Matrix  = 256 mm ×256 mm ×176 mm; FOV  = 290 mm ×290 mm ×176 mm, slice thickness  = 1.13 mm ×1.13 mm ×1.0 mm; Flip angle 20°) which was used to facilitate the spatial normalisation of the functional data.

### Stimuli

Eight digitized photographs were used from the NimStim set of facial expressions [38] of four males and four females each displaying a neutral expression. Stimulus presentation and data collection were controlled by E-Prime software [39].

### Training phase – outside the MRI scanner

Prior to entering the scanner, participants were trained to discriminate short (400 ms) from long (1,600 ms) stimulus durations. In the first block of 8 trials, a pink oval appeared for either a short or long duration in a fixed sequence (e.g., long-short-long-short, etc.). Participants were informed of this sequence and were asked to indicate whether the stimulus appeared for either a short or long stimulus duration by pressing one of two labelled keys on a QWERTY keyboard (the “z” and “m” keys were used). Following a response, visual feedback was given for both correct (“yes”) and incorrect (“no”) decisions. The feedback appeared in the centre of the screen for 2 seconds and was followed by an intertrial interval of 1200 msec. In second block of 8 trials, the pink oval was presented for a further 8 trials in a new random order for each participant. During this phase participants continued to indicate whether the oval appeared for either a short or a long stimulus duration. Participants continued to receive feedback in this phase. After the training phase participants completed the sex and time judgment tasks in the MRI scanner.

### fMRI design and procedure

Each scanning session consisted of 16 active blocks, each containing 10 trials, comprising 5 presentations of a single male face and 5 presentations of a female face. Active blocks were preceded and followed by passive rest blocks lasting 15 seconds. A mixed-model design was used: the type of task (Sex judgment, Time judgment) and identity of face was varied between blocks, while the duration of the faces (400, 700, 1000, 1300, 1600) and sex of face (male, female) were varied (on trial by trial basis) within each block. Specifically, on each trial the faces could vary in sex (either male or female) or duration, but always displayed a neutral expression. Each block was balanced so as to include 2 trials of each temporal duration in a random/pseudo-random order. All face stimuli were presented in a pseudo-randomized order within each block. The type of task was also randomised across blocks using a single, pseudo-randomized order. At the start of each block, a cue indicated whether participants should either judge the sex of the face as either male or female, or the judge the duration as either short or long. Participants were instructed to respond, using their dominant hand, via a button press, by pressing one button with their right index finger (for male and short) and another button with their middle finger (for female and long). Each face was separated by a fixed 1200 ms interval during which the screen appeared blank and participants made their response.

We attempted to minimise motor demands during the Sex and Time task by making participants delay their response until after the face had disappeared. Specifically, participants were instructed to respond after the offset of the face, during which the screen appeared blank for 1200 ms.

## Results

### Behavioural data analyses

The bisection point (BP) and Weber ratio (WR) were calculated for each participant, separately. The BP refers to the point of subjective equality (.5 point on the psychometric function) and was calculated using the method of least squares from the intercept and slope parameters of regression of p(long) onto stimulus duration [40]. The BP is an index of the perception of time that can be used to compare either relative under- or over-estimation of time across conditions [41], [42] or, as in the current research, relative differences in the perception of time between individuals [36]. A relatively low BP value (compared to other conditions or individuals) indicates that participants perceive more time as having passed; time is overestimated. The WR measures temporal sensitivity and is calculated by dividing half the difference between the upper difference limen (*p*long[.75]) and the and lower difference limen (*p*long[.25]) by the BP. In short, the BP is an index of perceived duration and the WR is an index of temporal sensitivity or precision.

### fMRI data modelling and analyses

Data were pre-processed and analyzed using SPM 8 software (Wellcome Trust Centre for Neuroimaging, London, UK). The initial 9 s of data (i.e. the first three volumes) from each scan session were removed to minimize the effects of magnetic saturation. Image data were analysed using a Mixed-Effects general linear model protocol in SPM8. At the first-level of analysis each participant's data were subjected separately to a regression analysis containing terms reflecting the predicted BOLD signal to the two active conditions and the passive baseline across the scanning session. These terms were produced by convolving a canonical haemodynamic response function with a box-car model representing the onset and duration of experimental blocks.

### Parametric modulation by stimulus duration

Also at the first-level, two further general linear models were created to assess the parametric modulation of BOLD signal by stimulus duration, on a trial-by-trial basis, using the first-order (linear) and second-order (quadratic) parametric modulation options integrated in SPM8. To isolate brain regions specifically engaged in the accumulation of time we modeled the linear (duration) by linear (task type) interaction that tested for regional BOLD signal change that rose linearly with stimulus duration (400, 700, 1000, 1300, 1600) that was greater (the slope was steeper) during Time judgment blocks compared to Sex judgment blocks. To test for activation associated with the difficulty of comparing the to-be-timed duration with the standard short and long durations (learnt previously) we modeled (using the 2^nd^ order parametric modulation option in SPM8) the quadratic (duration) X linear (task type) interaction that tested for regional BOLD signal change following a quadratic pattern (−2+1+2+1−2) that was greater during the Time judgment blocks compared to Sex judgment blocks. The latter analysis was based on the *a priori* assumption that difficulty would be greatest at 1000 ms where both response alternatives are likely to be weighted equally.

Predictors describing effects-of-no-interest included 6 regressors describing head-motion transformations that had occurred across each run, in order to remove residual fluctuations in the BOLD signal arising from such motion. Statistical parametric maps were generated for the contrast Time>Sex and also, the regression models constructed to test the differential linear (duration) by linear (task type) trend and the quadratic (duration) by linear (task type) trend. These were entered into a second-level t-test. A cluster forming height threshold corresponding to *t* = 7.26 was applied to the resulting contrast images, and clusters of activation that survived a cluster-level FWE-correction of p<.05 [43] were considered to be significant. Approximate anatomical labels for regions of significant clusters were determined using the SPM Anatomy toolbox version 1.8 [44]–[46].

### Individual differences – correlation analyses

For each individual, averaged beta parameters for BOLD activity across each cluster (where cluster ROIs were defined on the basis of the group-level analyses for the contrast Time>Sex) were extracted using MarsBar [47]. To test for individual differences, we computed correlations between the indices of timing (the BP and WF) and these beta parameters (alpha was set to. 05).

### fMRI results – Group

**Figure 1:**
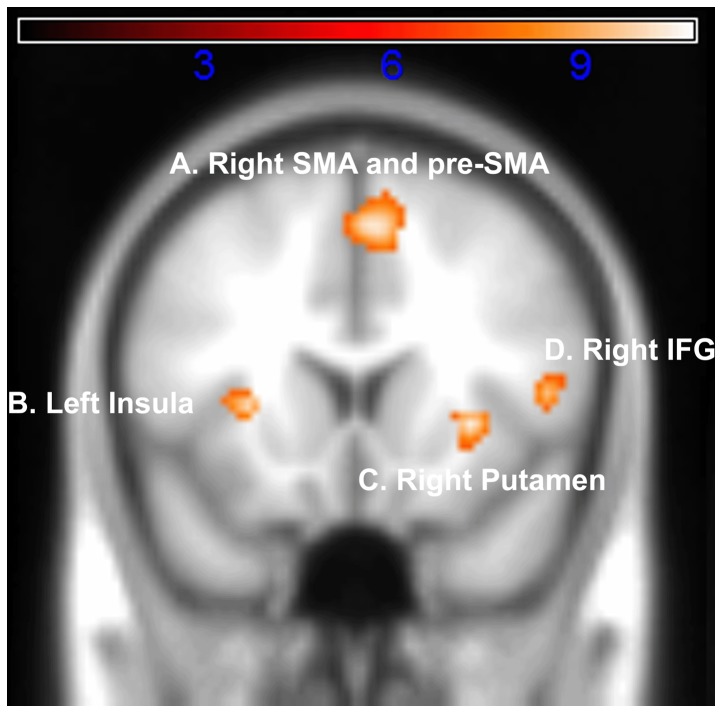
Coronal section (x = 50, y = 72, z = 47) of the significant activation for the Time>Sex contrast. Functional data are thresholded at *p*<0.05 (*t*>7.26) corrected for multiple comparisons using FWE and are superimposed as a colour overlay on the average of 152 anatomical scans from different brains (Montreal Neurological Institute). *Key* A. Right SMA (MNI: x = +4, y = +16, z = +52) and Right pre-SMA (MNI: x = +6, y = +26, z = +48). B. Left Insula (MNI: x = −28, y = +18, z = +4). C. Right Putamen (MNI: x = +30, y = +16, z = 0). D. Right Inferior Frontal Gyrus (pars opercularis) (MNI: x = +50, y = +14, z = +8).

The SPM t-test for the contrast Time>Sex revealed 7 FWE-corrected clusters which are described in Table 1. Four of these clusters are shown in Figure 1, in which active regions identified in the group analysis are superimposed on the MNI-152 template brain used for the spatial normalization. For the contrast Time>Sex there was greater activation in both the right pre-SMA and right SMA (Cluster 1), right Putamen (Cluster 2), left Insula (Cluster 3), Right Inferior Frontal Gyrus (pars opercularis) (Cluster 4), Right Middle Frontal Gyrus (Cluster 5), Left Inferior Frontal Gyrus (pars opercularis) (Cluster 6) and Right Pallidum (Cluster 7).

**Table 1:** Areas of significant activation at the threshold *p*(FWE) <.05 (cluster-level corrected) for the contrast Time>Sex.

Voxel No.	*X*	*Y*	*Z*	*t* value	Location	*r* (BP)	*r* (WR)
239	4	16	52	10.76**	Right SMA	−.71*	.27
	6	26	48	10.51**	Right Pre-SMA		
165	30	16	0	10.40**	Right Putamen	−.1	.48
49	−28	18	4	10.29**	Left Insula	−.44	.29
43	50	14	8	8.64**	Right IFG (pars opercularis)	−.72*	.37
21	34	40	24	8.44**	Right Middle Frontal Gyrus	−.35	−.41
9	−48	10	8	8.25**	Left IFG (pars opercularis)	−.39	.67*
2	18	8	−2	7.65**	Right Pallidum	−.37	.09

MNI coordinates indicate the peak voxel (*t*>7.26) within each anatomical region. The final two columns display the Pearson product moment correlation coefficients (*r*) between both the Bisection Point (BP) and Weber Ratio (WR) and activity in each of the regions identified as more active for the contrast Time>Sex.

* *p*<.01.

** cluster-level *p*(FWE) <.05.

Abbreviations: SMA  =  Supplementary Motor Area; IFG  =  Inferior Frontal Gyrus.

**Figure 2:**
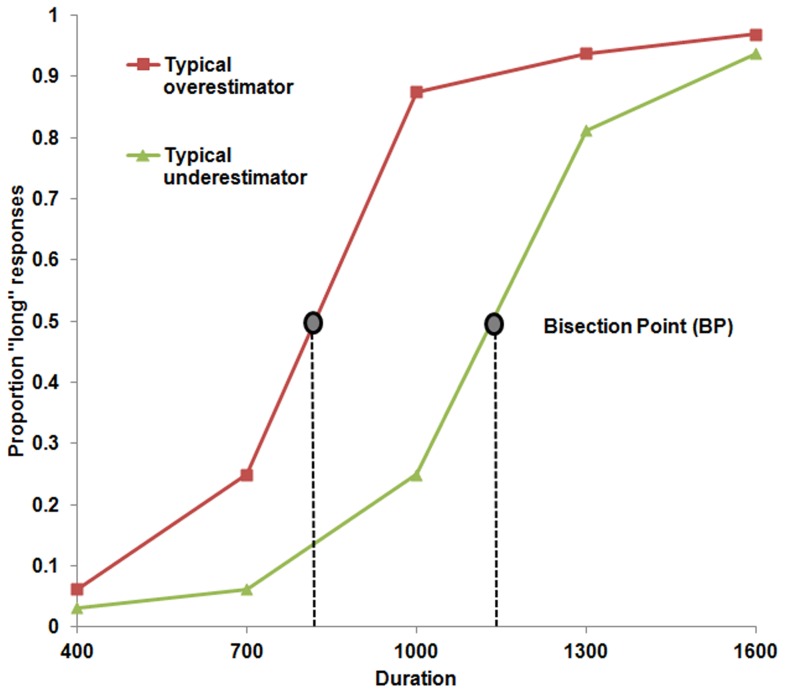
Psychometric curve of the mean proportion of ‘long’ responses as a function of stimulus duration for a typical underestimator (relative to the group-averaged BP) and a typical overstimator (relative to the group-averaged BP). The BP (duration at which participants respond equally often short and long) for each individual is marked on each curve. Overestimation is reflected by a leftward shift in the BP and underestimation is reflected by a rightward shift.

fMRI activation and Bisection Point. A programming error meant that the behavioural data from 3 participants was not recorded and consequently, correlation coefficients were calculated based on the data of the remaining 14 participants. In keeping with other studies (for a meta-analysis see; [48]) where the spread or ratio of ‘‘long” and “short” reference durations is relatively large (≥4) the mean BP was closer to arithmetic mean (*M* = 974 ms; *SD*  = 112) than the geometric mean. The correlation coefficients that model the relationship between the BP and the increase in activation in each of the regions that were identified as more active in Time>Sex contrast are displayed in Table 1. Psychophysical curves representing the relationship between the proportion of long responses and the stimulus duration for a participant who underestimated the face duration (compared to the group-averaged BP) and an individual who overestimated the face duration are displayed in Figure 2. ). There was a significant negative correlation between the bisection points and the beta coefficients for activity (for the contrast Time>Sex) in the right SMA (*r* = −.71, *p* = .003; 95% CI for *r = *−.9 to −.3) and also between the BP and the beta coefficients for activity in the Right Inferior Frontal Gyrus (pars opercularis) (*r* = −.72, *p* = .002; 95% CI for *r = *−.9 to −.3). In other words, (since a low BP indexes a tendency to overestimate elapsed time) levels of BOLD activation in both right SMA and right pars opercularis were associated with increases in perceived duration. That is, activity in these regions indexes the subjective perception of duration as well as the actual duration.

**Figure 3:**
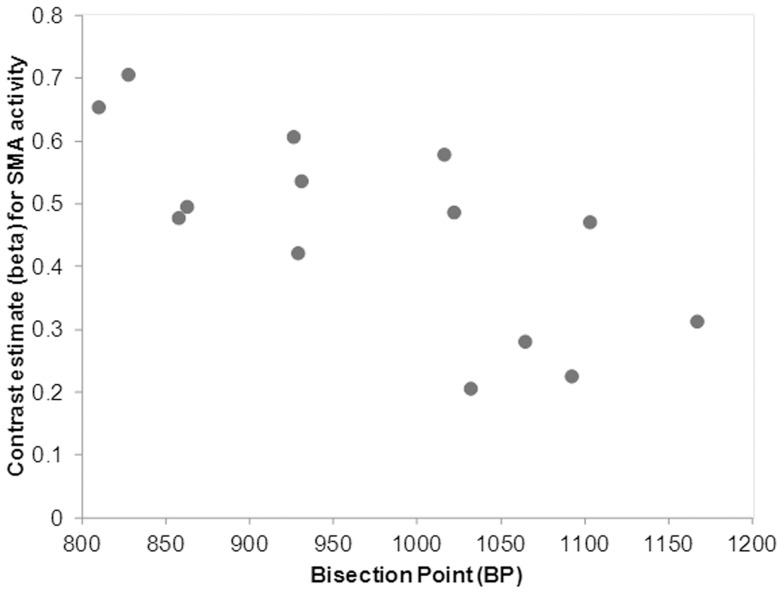
Scatterplot displaying relationship between mean Bisection Point for each individual and mean individual activation (beta values) for the SMA for the contrast Time>Sex.

**Figure 4:**
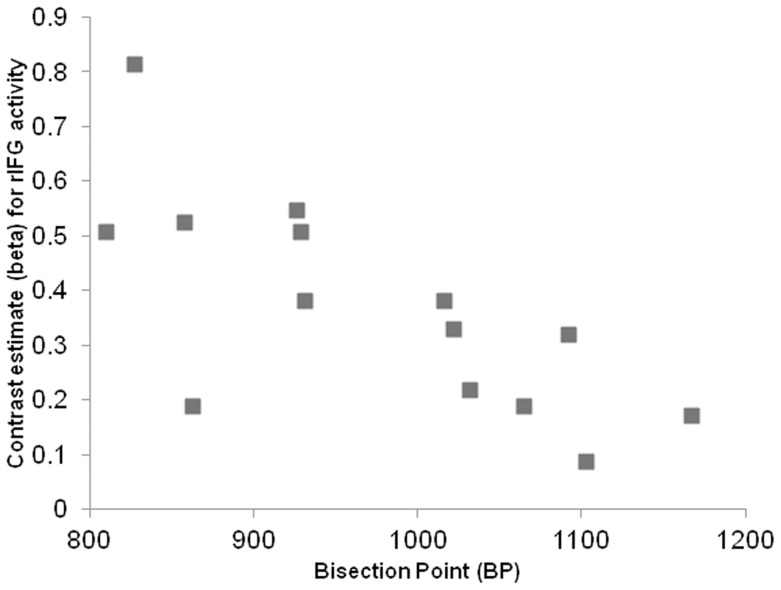
Scatterplot displaying relationship between mean Bisection Point for each individual and mean individual activation (beta values) for the right inferior frontal gyrus (rIFG) for the contrast Time>Sex.

The correlation between the mean BP for each individual and mean activation (beta values) in the (for each individual), is displayed in a scatterplot in Figure 3. A scatterplot displaying relationship between mean BP for each individual and mean individual activation (beta values) for the SMA for the contrast Time>Sex is displayed in Figure 4. A scatterplot displaying relationship between mean BP for each individual and mean individual activation (beta values) for the right inferior frontal gyrus (rIFG) for the contrast Time>Sex is displayed in Figure 4. All other correlations were negative but smaller in magnitude (largest *r* = −.44) and failed to reach statistical significance.

fMRI activation and Weber index. The mean WR was. 22 (*SD*  = .04). The correlation coefficients for the relationship between the WR and the increase in activation in each of the regions identified as more active in Time>Sex contrast are displayed in Table 1. There was a significant positive correlation between the WR and the beta coefficients (for the contrast Time>Sex ) for activation in the Left Inferior Frontal Gyrus (pars opercularis) (*r* = .67, *p* = .007; CI −.9 to −.3In other words, temporal sensitivity decreased with increases in activation in the Left Inferior Frontal Gyrus. All other correlations were smaller in magnitude (largest *r = *.48) and failed to reach statistical significance.

### Parametric modulation by stimulus duration

Neither the linear (duration) by linear (task type) interaction nor the quadratic (duration) X linear (task type) interaction resulted in significant clusters of activation (using *p*<0.05 FWE-corrected for multiple comparisons across the whole brain). In light of the possible reduction in signal-to-noise ratio associated with these analyses [49], which are based on trial-by-trial fluctuations in the BOLD signal, we attempted to increase power to detect relevant effects testing for parametric modulation within the ROIs identified in the main analyses by using the Time>Sex contrast image as an inclusive mask. In other words, we restricted our test for parametric modulation to the areas identified as significant for the Time>Sex contrast. For the linear by linear contrast there was voxel level activation (*p*<.05 FWE-corrected across all voxels in the mask) in the SMA. The quadratic by linear contrast was not significant in any of the ROIs.

## Discussion

Our task revealed highly consistent activation in the major areas identified in previous research [3] as responsible for time perception. Specifically, we recorded activation in the basal ganglia including the putamen, insula and right pallidum and, in agreement with a recent meta-analysis [26], left and right inferior frontal gyrus and SMA. A further striking finding was that individual differences in timing behaviour predicted activation in the neural regions identified both here, and separately [50], as responsible for timing. Specifically, participants who perceived time as lasting longer (as indexed by lower bisection values) showed greater activation in both the rIFG and SMA.

The findings can be interpreted within the context of internal clock models of timing [9]. Two aspects of our data support the idea that the SMA is responsible for the accumulation of units of time. First, the subjective lengthening of perceived time as indexed by a leftward shift in the BP (indicating greater relative overestimation) predicted increases in activation in the SMA and rIFG. This result would be expected if the SMA were to act as an accumulator because perceived time is assumed to lengthen with the passage of time, as more units of time enter the accumulator. Second, in keeping with results of recent research [10], [11], our parametric analyses showed that activation in the SMA was predicted by a linear but not quadratic pattern of changes in stimulus duration. These results are in keeping with the idea of an accumulator because linear increases in activation are expected to co-occur with changes in both the subjective experience of time and the actual duration. A role for the SMA in the accumulation of time is consistent with other neuroimaging studies that have recorded increased activation in the SMA with increases in range of durations used [50] and also parametric modulation of SMA activation due to linear increases in both attention to time [5] and stimulus duration [10]. Moreover, in a similar vein to the effects reported here, one study [2] showed that individual differences in timing performance predicted activation in the SMA. Specifically, in the latter study, there was relatively greater activation in the SMA for strong compared to weak beat perceivers.

It is important to note that the findings of previous research and those reported here, do not permit the conclusion that the SMA is solely responsible for temporal accumulation. For example, in the current study, we found that activation in the rIFG correlated with linear increases in the subjective experience of time. Similarly, other studies [7], [10], [11] that have tested for an accumulation of neural activity due to time have reported effects across several brain regions. For example, in the research reported by Bueti and colleagues [24] there was a linear relationship between temporal reproduction times (as an index of the subjective perception of time) and activity across a number of regions (putamen, insula and mid/superior temporal gyrus) during the temporal encoding of a visual but not an auditory stimulus. The latter finding agrees with considerable evidence [11] that the basal ganglia (including the putamen) play a key role in interval timing [51] and consequently, it has been argued [52] that the neural clock resides in the basal ganglia. Given that the basal ganglia and SMA are highly connected neural structures, attempts to dissociate the processes associated with these neural structures remains a challenge for neuroscientists. Again, manipulations that target the operation of specific mechanisms within the internal clock [53] offers one method to help disentangle the role of different neural structures. A second useful approach is to model the sequence of activation from one structure to another using statistical techniques such as dynamic causal modelling [54]. In short, although further research is needed, our data and the results of other research tentatively support the idea that the SMA is a key neural structure in temporal accumulation.

A notable feature of the current research is that large, statistically reliable neural activation was recorded in the neural regions previously found to be active during temporal discrimination using a standardised test of temporal performance and a relatively small sample size. Furthermore, the fMRI protocol lasted less than 10 minutes. Given the high cost of fMRI research, these features mean that further research into the effects recorded here will most likely be relatively inexpensive to replicate using a relatively small sample (*n*<18). Nonetheless, to assess the relative contribution of individual differences in timing performance across all the neural areas more active during time discrimination (compared to the control task) would still require a relatively large sample to avoid Type II errors [55]. This means that it would be a mistake to conclude from the non-significant correlations that specific regions do not perform timing-related functions.

In addition to the predicted association between timing performance and activity in the SMA there was also a significant positive correlation between timing sensitivity measured by the Weber ratio (WR), and activity in the Left Inferior Frontal Gyrus. Higher WR values indicate decreased temporal sensitivity and therefore this effect shows that activity in the Left Inferior Frontal Gyrus is associated with decreased temporal sensitivity. Activity in the Left Inferior Frontal Gyrus (LIFG) increases with response selection demands [56] and therefore, one explanation for this correlation is that LIFG activity reflects individual differences in the ability to select either short or long response (and subsequent decreased temporal sensitivity). Put differently, the harder the decision to select between the short and long response options (and consequent poorer sensitivity), the greater the activation in the LIFG. This hypothesis was not supported by our modelling of a quadratic effect of stimulus duration. We tested the quadratic trend based on the assumption that difficulty would be greatest at 1000 ms where both response alternatives are likely to be weighted equally. However, caution needs to be taken when interpreting the these group-averaged null effects because they are restricted by a reduction in the signal to noise ratio for our trial-by-trial analyses [49]. Therefore, it is advisable that future research use research design features that enhance signal-to-noise ratio for mixed designs, such as “jittering” inter-trial intervals according to a uniform distribution.

The current study opens several fruitful avenues for further research. For example, we intend to establish how emotion modulates the mechanisms responsible for timing by varying the facial expression on the face. Previous research [33] has shown that participants overestimate the duration of emotionally arousing facial expressions relative to neutral expressions. Such findings have been interpreted as reflecting the speeding of an arousal-sensitive pacemaker that resides within an internal clock [57]. Using our task it will be possible to elucidate the key neural mechanisms engaged during both timing and the modulation of timing due to emotion. Of particular interest is the link between activation in specific limbic structures such as the amygdala, that are typically active during the processing of emotional stimuli [12] and activation in the core structures within the timing network. Furthermore, the task may also be of use to researchers wishing to study the role of timing processes in motor simulation of facial expressions. The effects of emotion on time are eliminated when participants are prevented from mimicking (and therefore, simulating) the facial expression of the person they are observing [58]. Moreover, there is considerable overlap between the inferior frontal and pre-motor regions responsible for timing reported here and those reported elsewhere [59], [60] as engaged in the simulation of facial expressions and therefore, one hypothesis is that timing during social interaction depends on the neural regions responsible for motor simulation.

In summary, in addition to providing converging evidence for the SMA and Right Inferior Frontal Gyrus as key neural regions that underpin time discrimination, the current study describes a short, simple task that is sensitive to detecting activity associated with individual differences in subjective estimates of time. The important point raised here is that individual differences in time perception may play a considerable role in understanding the neural processes that underlie time perception.
